# Differences Between Two Devices With Function of Periopolishing and Piezoelectric Scaler: A Randomized Clinical Trial

**DOI:** 10.1155/ijod/7131637

**Published:** 2025-03-04

**Authors:** Alessandro Chiesa, Maurizio Pascadopoli, Andrea Butera, Marco Monticone, Maria Mirando, Filippo Vezzoni, Andi Kertalli, Andrea Scribante, Gianna Maria Nardi

**Affiliations:** ^1^Unit of Dental Hygiene, Section of Dentistry, Department of Clinical, Surgical Diagnostic and Pediatric Sciences, University of Pavia, Pavia 27100, Italy; ^2^Unit of Orthodontics and Pediatric Dentistry, Section of Dentistry, Department of Clinical, Surgical, Diagnostic and Pediatric Sciences, University of Pavia, Pavia 27100, Italy; ^3^Department of Surgical Sciences, University of Cagliari, Cagliari, Italy; ^4^Unit of Periodontology, Section of Dentistry, Department of Clinical, Surgical, Diagnostic and Pediatric Sciences, University of Pavia, Pavia 27100, Italy; ^5^Department of Oral and Maxillofacial Sciences, Sapienza University of Rome, Via Caserta 6, Rome 00161, Italy

**Keywords:** air polishing, comfort operator, dental hygiene, dentistry, oral care, periodontal parameters, periodontology, piezoelectric scaler, randomized clinical trial, ultrasound

## Abstract

Dental procedures often cause anxiety and apprehension in patients due to potential discomfort and pain. Understanding patient sensitivity and comfort is critical to providing effective dental care. The aim of this study was to compare patient and operator comfort between two devices with the function of periopolishing and piezoelectric scaler during guided biofilm therapy (GBT) sessions. A randomized controlled trial was conducted involving 20 patients and operators. Primary outcomes, including pain, sensitivity, and operator comfort, were assessed using a visual analog scale (VAS), while periodontal indices served as secondary outcomes. The results showed comparable levels of patient and operator comfort between the two devices, with higher airflow comfort reported for the Mectron Combi Touch and higher ultrasonic handpiece comfort showed for the EMS Prophylaxis Master. Both devices effectively reduced periodontal parameters without significant differences. These results indicate that both Mectron Combi Touch and EMS Prophylaxis Master offer viable options for efficient and patient-friendly periodontal treatment. The study provides valuable insights for improving patient experience and treatment outcomes in the dental office. Considerations for device selection based on clinical needs and patient comfort are discussed, as well as future perspectives for advancing preventive dental care. This research contributes to the ongoing topic of improving dental procedures to ensure optimal patient satisfaction and oral health outcomes.

**Trial Registration:** ClinicalTrials.gov identifier: NCT05263622

## 1. Introduction

Dental procedures often evoke feelings of anxiety and apprehension in patients, primarily due to the potential for pain and discomfort. Dental sensitivity, encompassing both physical sensations and psychological responses, plays a crucial role in shaping patients' experiences during dental treatments [1]. Understanding the interplay between these factors is crucial for providing compassionate and effective dental care [2].

Research suggests that patient experiences and perceptions of pain during dental procedures can significantly influence their satisfaction with dental care and willingness to seek treatment in the future. Factors such as fear, past traumatic experiences, and individual pain thresholds contribute to variations in sensitivity among patients. Additionally, cultural and socioeconomic backgrounds may influence how patients perceive and express discomfort during dental visits [3]. Recognizing and addressing these nuances enhances patient trust, satisfaction, and treatment adherence [4, 5]. Technological advancements in modern dentistry have introduced innovative tools designed to improve patient outcomes and operator efficiency. Among these, air polishing with powders such as erythritol and glycine, in combination with ultrasonic scalers, has become a cornerstone in periodontal therapy and oral hygiene maintenance [6, 7]. This synergistic approach addresses both supragingival hygiene and subgingival debridement, catering to a diverse spectrum of dental needs with precision and efficiency [8, 9]. The escalating adoption of air polishing techniques reflects a growing body of evidence supporting their effectiveness in plaque removal, calculus disruption, and biofilm management, thereby mitigating the risk of periodontal diseases and promoting optimal oral health outcomes [10]. Studies have underscored the superior plaque removal capabilities of air polishing with erythritol and glycine powders compared to traditional methods, attributing it to the gentle jet thorough nature of the process [11]. The use of ultrasonic scalers has demonstrated benefits in subgingival debridement, including enhanced access to periodontal pockets, efficient removal of calculus, and reduced physical strain on the operator [12]. This confluence of innovations underscores a paradigm shift in dental practice, emphasizing a proactive approach to periodontal care and the management of oral diseases.

These cutting-edge instruments represent a significant departure from traditional dental tools, offering precise and efficient solutions for various oral care procedures. Understanding the nuances and disparities between devices incorporating air polishing and scaler functionalities, both driven by piezoelectric mechanisms [13], is paramount for dental professionals seeking to optimize treatment modalities and patient experience [14]. The Mectron Combi Touch and EMS Prophylaxis Master exemplify such cutting-edge technologies, integrating air polishing and piezoelectric ultrasonic scaling functionalities. These devices embody a shift in dental practice toward a proactive, patient-centered approach to periodontal care. The Mectron Combi Touch combines piezoelectric scaling with advanced air-polishing capabilities, allowing precise adjustment of parameters for different clinical scenarios. Meanwhile, the EMS Prophylaxis Master incorporates guided biofilm therapy (GBT), featuring an ergonomic design and optimized workflow aimed at enhancing operator comfort and patient experience. Exploring the distinctions between these devices in terms of performance, patient comfort, and operator usability is critical for optimizing clinical outcomes.

Despite the growing interest in patient comfort assessments, there is a notable lack of studies in the literature that specifically utilize and compare instruments like those employed in this study. Many existing works focus on individual tools or scales but do not provide a direct comparison, leaving a gap in understanding their relative efficacy. This lack of comparative research motivated the design of our study, which aims to fill this gap by evaluating and contrasting the performance of different instruments. By addressing this underexplored area, our study seeks to provide valuable insights into the use of these tools in clinical settings.

Based on these considerations, the objectives of the present study were to evaluate the timing and comfort of the patient and operator using the following equipment for a GBT session: EMS Prophylaxis Master and Mectron Combi Touch. The null hypothesis of the study was that no significant difference was found between the subjective perceptions of the two devices, both from the patient's perspective and the operator's one.

## 2. Materials and Methods

### 2.1. Trial Design

This was a randomized clinical trial with a split-mouth design. An equal enrollment of patients was adopted. The study was approved by the Unit Internal Review Board (registration number: 2022-0216).

### 2.2. Participants

The study was conducted at the Unit of Dental Hygiene, Section of Dentistry, Department of Clinical, Surgical, Diagnostic and Pediatric Sciences of the University of Pavia, Italy. The study started in March 2022 and ended in September 2022. Patients were asked to sign the informed consent before participating. Interventions and outcomes assessment were conducted at the same unit.

The patients included in the study had periodontal disorders, in maintenance therapy, attributable to Grade A or B and Staging I-III of the new Classification of Periodontal and Peri-Implant Diseases and Conditions [15, 16].

The inclusion and exclusion criteria are shown in Table 1.

### 2.3. Interventions and Outcomes

At the first meeting (baseline, T0), after having adequately and fully explained the purpose of the study, the patient signed the informed consent. Then the biographical and anamnestic data, dental and periodontal charting were collected. The patients were visited, and the following epidemiological indices of oral health [17] were collected by an instructed operator using a probe (Table 2): bleeding on probing (BoP) [18], bleeding score (BS) [19], and plaque control record (PCR) [20].

Afterward, patients were randomly assigned to two groups according to the split-mouth design of the study; in group 1, patients underwent:

• Collection of periodontal indexes (BoP, BS, PCR).
• Plaque detector use (Mira 2 ton tablets, Hager & Werken Duisburg, Germania) to show the patient where most plaque is located.
• Motivation for correct home oral hygiene techniques.
• Deplaquing by airflow EMS (Q1 and Q4 quadrant).
• Calculus removal using EMS ultrasonic handpiece (Q1 and Q4 quadrant).
• Deplaquing using Mectron Combi Touch airflow (Q2 and Q3 quadrant).
• Calculus removal using Mectron Combi Touch ultrasonic handpiece (Q2 and Q4 quadrant).
• Checking for soft-tissue residues (plaque) and hard-tissue residues (calculus).

In group 2, quadrants were inverted. At the end of the GBT protocol treatment of two antagonistic hemiarches, the patient was subjected to a questionnaire with questions regarding the oral hygiene session. Afterward, the remaining two hemiarches were treated with the same methods, and, again, the patient was subjected to a questionnaire with questions concerning the oral hygiene session. The only request from the operator to the patient was to pay close attention to individual sensations during the oral hygiene session. The characteristics of the devices used in the study are shown in Table 3.

The primary outcomes we investigated were pain and sensitivity perceived by the patient, assessed with a visual analog scale (VAS) scale, and operator comfort assessed by questionnaire with a 0–10 scale after using the two devices. The operator questionnaire aimed to explore, in addition to the comfort of ultrasound (US) and airflow, variables such as the time for the session and the number of droplets assessed for both instruments. The periodontal indexes (BoP, BS, PCR) were considered secondary outcomes. The patients returned to the clinic after 30 days (T1) for a re-evaluation of the periodontal indexes. The erythritol powder that was used in the two airflow handpieces (EMS, Mectron) was weighed before use and after finishing the treatment, and the time of use of each handpiece was timed so that the powder consumption in minutes of use could be estimated.

### 2.4. Sample Size

Sample size calculation was conducted (Alpha = 0.05; Power = 95%) considering two independent study groups. The following mathematical formula was used for sample size calculation:

 

  $$\begin{array}{c} \text{Sample size}=\frac{Z_{\left(1-\frac{\alpha }{2}\right)}^{2}p\left(1-p\right)}{d^{2}}, \end{array}$$

where *Z* is the standard normal variate corresponding to 1.96 at 5% type 1 error, *p* is the expected proportion in population expressed as decimal and based on previous studies, and finally, *d* is the confidence level decided by the researcher and expressed as decimal, too. The primary outcome considered was “Pain” through VAS. An expected mean of 9.3 and an expected difference between the means was supposed to be 7.3 with a standard deviation of 10.9 [9]; therefore, 40 quadrants per group were required for the split-mouth study.

### 2.5. Randomization

The study employed a split-mouth design to enhance internal validity by minimizing patient-level confounding factors. Each participant underwent both the experimental and control treatments in different quadrants of the same mouth, facilitating a direct comparison within the same individual while accounting for variations in anatomy, baseline conditions, and treatment responses [21].

Treatment allocation followed the SNOSE method (opaque, sequentially numbered envelopes), which provided additional protection against allocation bias. Block randomization was employed using a block randomization table involving a permuted block of 80 quadrants. Once one quadrant was assigned for Mectron treatment, the opposite quadrant was assigned to EMS treatment.

Blinding was implemented for both participants and data analysts to ensure an unbiased evaluation of outcomes. A single operator performed all procedures and data collection after quadrant assignment.

### 2.6. Statistical Methods

Data underwent statistical analysis with R Software (R version 3.1.3, R Development Core Team, R Foundation for Statistical Computing, Wien, Austria). Mean, standard deviation, minimum, median, and maximum were calculated for each group and variable as descriptive statistics. The Shapiro–Wilk test was performed for data normality assessment. For the VAS scales for operator and patient perceptions, the Mann–Whitney *U* test was performed, while for periodontal indexes, ANOVA followed by Tukey's post hoc test was performed. Significance was predetermined at *p* < 0.05 for all the tests performed.

## 3. Results

### 3.1. Participant Flow and Baseline Data

Participants were enrolled until the required number of quadrants was not reached. In total, 20 patients were enrolled according to the inclusion criteria; they agreed to participate and received the allocated interventions; 20 operators were involved in the evaluation of the two devices used in the study.

No patient or operator was excluded from the analysis.

### 3.2. Primary Outcomes

**Figure 1:**
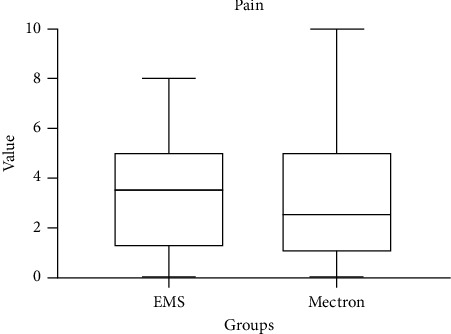
Comparison of pain scores between EMS and Mectron following the treatment (0: no pain; 10: maximum pain).

**Figure 2:**
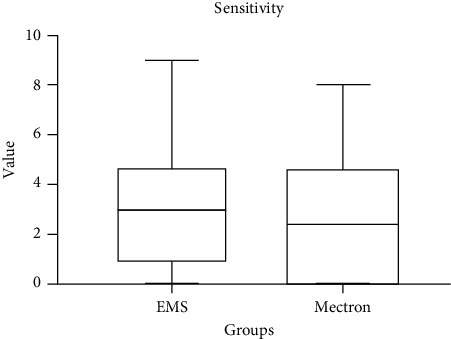
Comparison of sensitivity scores between EMS and Mectron following the treatment (0: no sensitivity; 10: maximum sensitivity).

**Figure 3:**
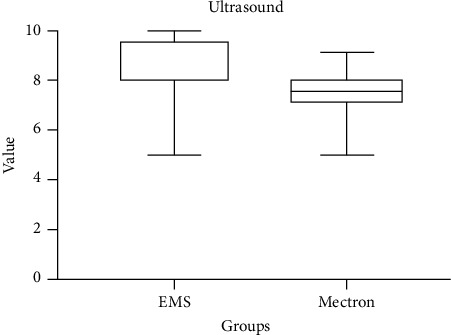
Comparison of ultrasound comfort scores between EMS and Mectron following the treatment (0: no comfort; 10: maximum comfort).

**Figure 4:**
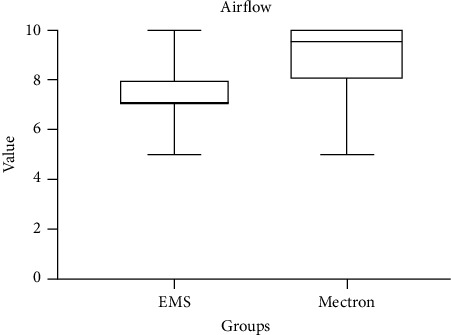
Comparison of airflow comfort scores between EMS and Mectron following the treatment (0: no comfort; 10: maximum comfort).

Primary outcome scores detected at T0 are shown in Table 4. The variable airflow comfort (*p* < 0.05) showed higher values for the Mectron Combi Touch device. On the other hand, concerning US comfort, EMS Prophylaxis Master showed significantly higher values (*p* < 0.05). As regards other outcomes, no significant differences between groups were detected (*p* > 0.05) (Figures 1–4).

Box plots comparing perceived comfort during treatment with two different instruments: US and airflow. The data show distinct distributions of comfort levels, with significant variations between the two instruments. Median values, interquartile ranges, and potential outliers are displayed, providing insights into patient-reported comfort for each method.

### 3.3. BoP

BoP scores recorded at T0 and T1 are shown in Table 5. A significant decrease was found in both groups after 30 days (*p*  < 0.05). Intragroup comparisons showed significant differences in the intervals T0–T1 in both groups (*p* < 0.05). No significant intergroup difference was found at T1.

**Figure 5:**
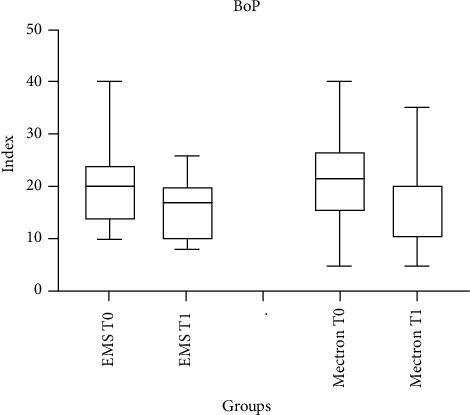
Bleeding on probing (BoP) measured over time (T0 vs. T1) after treatment with EMS and Mectron.

Figure 5 provides a clear comparison of the BoP index over time for the two instruments used in the study. Both instruments show a reduction in BoP posttreatment, with one demonstrating a more pronounced and sustained decrease. These findings underline the effectiveness of the instruments in reducing gingival inflammation and bleeding, with differences potentially attributable to their specific mechanisms of action.

### 3.4. PCR

PCR percentages recorded at T0 and T1 are shown in Table 6. Significant intragroup differences were found in both groups between T0 and T1 (*p* < 0.05), while no significant differences between groups were found at T1 (*p* > 0.05).

**Figure 6:**
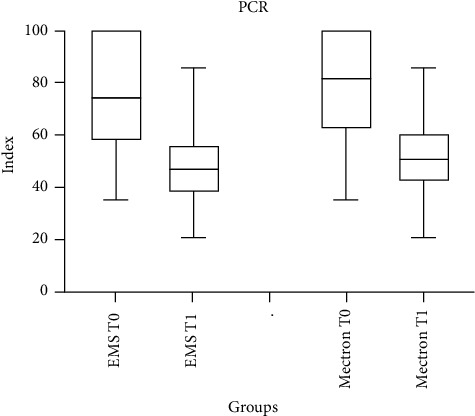
Plaque control record (PCR) measured over time (T0 vs. T1) after treatment with EMS and Mectron.

Figure 6 presents the changes in the PCR from baseline (T0) to posttreatment (T1) for the two instruments. Both demonstrate a reduction in plaque levels, with one instrument achieving a more pronounced improvement. These findings underline the effectiveness of the instruments in improving plaque control, providing valuable insights into their clinical performance and patient outcomes.

### 3.5. BS

BS values recorded at T0 and T1 are shown in Table 7. Intragroup comparisons showed a significant decrease at the interval T0-T1 in both groups (*p* < 0.05). Intergroup comparisons highlighted did not show a significant difference between the groups at T0 and T1 (*p* > 0.05).

**Figure 7:**
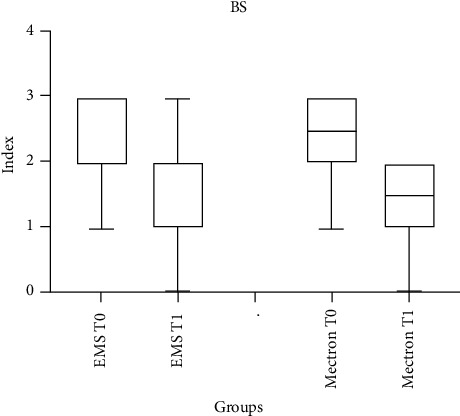
Bleeding score (BS) measured over time (T0 vs. T1) after treatment with EMS and Mectron.

Figure 7 shows the BSs at two time points, T0 and T1, for both instruments used. For both instruments, the BS values at T1 are lower compared to T0, indicating a reduction in BSs over time. This suggests that the intervention or treatment may have had a positive effect in reducing bleeding, as reflected by the lower scores at T1 across both instruments. The trend of decreasing scores provides insight into the effectiveness of the approach being evaluated.

## 4. Discussion

In recent years, advancements in technology have introduced innovative devices to aid dental professionals in their practice. Two such devices, the Mectron Combi Touch and the EMS Prophylaxis Master, have gained attention for their effectiveness in dental prophylaxis.

Then null hypothesis of the study was partially accepted. No significant differences were found between VAS scores on personal assessments of patients during the administered treatments. This indicates that while the devices perform similarly in most aspects, certain slight differences may influence clinical decision-making, particularly in terms of comfort and sensitivity.

The results of this study show that, on average, the Mectron Combi Touch for patients is considered slightly less painful than the EMS Prophylaxis Master and causes slightly less sensitivity according to VAS scores, even if these differences are not significant. Operators had different sensations; in fact, the EMS Prophylaxis Master, with its handpieces, compared to the Mectron Combi Touch, was found to be significantly more comfortable to use, with an average EMS airflow comfort of 13.8% higher than the Mectron airflow. As regards the ultrasonic handpieces, operators found the EMS ultrasonic handpiece 7.6% significantly more comfortable. The BoP in 20 patients decreased from T0 to T1 by an average of 3.95% for the Mectron Combi and 3.75% for the EMS Prophylaxis Master, and the BS went from an average Mectron Combi Touch value of 2.3 to an average of 1.35 in 30 days, while the EMS Prophylaxis Master goes from an average value of 2.25–1.45 in 30 days.

Regarding the PI, there is a significant decrease for both machines, with an average decrease from T0 to T1 of 27.5% for the Mectron Combi Touch and 29.5% for the EMS Prophylaxis Master.

In summary, the subjective variables showed higher airflow comfort reported for the Mectron Combi Touch and higher ultrasonic handpiece comfort showed for EMS Prophylaxis Master. The results showed that the variation in periodontal indices is almost similar in both machines. Periodontal variables decreased in both groups, with no significant between-group differences.

The slight differences in airflow comfort and ultrasonic handpiece use suggest that these devices may be better suited to different patient populations. For instance, patients with heightened sensitivity might benefit from the Mectron Combi Touch, while operators who prioritize ergonomic handling might prefer the EMS Prophylaxis Master.

Air polishing and piezoelectric scaling are two commonly used techniques in dental prophylaxis, each with its own advantages and limitations. Air polishing involves the use of a high-pressure stream of air, water, and abrasive powder to remove stains, plaque, and biofilm from teeth surfaces. This method is highly effective in achieving quick and efficient stain removal, making it popular for patients seeking esthetic improvements. However, air polishing may not be suitable for patients with respiratory conditions due to aerosol generation [22, 23]. Studies have shown that air polishing is highly effective in removing extrinsic stains and plaque from tooth surfaces, resulting in a significant improvement in the cleanliness and smoothness of teeth [24]. Furthermore, research indicates that air polishing can achieve superior results compared to traditional polishing methods, such as rubber cup polishing, particularly in terms of stain removal and surface smoothness [25]. One study found that air polishing with glycine powder resulted in a greater reduction in plaque and gingival inflammation compared to traditional hand scaling and polishing [26]. Another study demonstrated that air polishing effectively removed biofilm from tooth surfaces, leading to improved oral hygiene and reduced risk of periodontal disease [27]. Moreover, patient satisfaction with air polishing is consistently high due to its gentle nature and minimal discomfort during the procedure. Patients often report immediate improvements in the brightness and cleanliness of their teeth following air polishing, leading to increased confidence in their smile and overall oral health [28].

On the other hand, piezoelectric scaling utilizes ultrasonic vibrations to break down and remove calculus deposits from teeth surfaces. Research indicates that piezoelectric scalers effectively remove calculus and plaque from tooth surfaces, leading to improved periodontal health and reduced risk of periodontal disease [29]. Piezoelectric scalers are also generally gentler on the tooth surface compared to traditional hand scalers, reducing the risk of enamel damage and patient discomfort. However, they may be less effective in stain removal compared to air polishing [30, 31]. Compared to traditional scaling methods, such as hand scaling, piezoelectric scalers offer several advantages, including greater precision and efficiency in calculus removal [32]. Studies have demonstrated that piezoelectric scalers generate less heat and vibration compared to ultrasonic scalers, resulting in reduced discomfort for patients during the scaling procedure [33]. Additionally, the ultrasonic vibrations produced by piezoelectric scalers have been found to disrupt biofilm and bacterial colonies on tooth surfaces more effectively, leading to improved oral hygiene outcomes [34]. Furthermore, patient satisfaction with piezoelectric scaling is consistently high due to its gentle and efficient nature [35].

Despite these promising results, the study has some limitations that warrant consideration. For instance, the reliance on patient-reported pain assessments introduces the potential for bias, and the short-term follow-up limits the ability to assess long-term clinical outcomes. Future studies should address these limitations by incorporating objective measures of patient discomfort and extending follow-up periods to evaluate the durability of treatment effects.

Regarding the variables to be considered when choosing between the two instruments, some results can be deduced from the literature. Research studies have demonstrated the efficacy of ultrasonic scaling devices like the Mectron Combi Touch in plaque and calculus removal compared to traditional hand scaling methods. For example, a review by Oza et al.[36] found that ultrasonic scaling was more effective in calculus removal and less time-consuming than hand scaling, leading to better patient outcomes and satisfaction.

Several clinical studies have investigated the efficacy and safety of air polishing systems like the EMS Prophylaxis Master. For instance, a study by Petersilka et al. [37] compared air polishing with traditional rubber cup polishing and found that air polishing resulted in superior stain removal and surface smoothness, with reduced risk of enamel damage.

The results found from Simon et al. [38] study suggest that air scaling is reasonably effective in removing plaque and in reducing gingival index scores, although ultrasonic scaling produced better results in terms of plaque index scores in comparison. Air scaling has previously been shown to be as effective as ultrasonic scalers and curettes in removing subgingival biofilm in periodontal pockets with probing depths of up to 4 mm [39]. Wennström, Dahlén, and Ramberg [26] conducted a study to determine the clinical and microbiological effects of subgingival air polishing and ultrasonic instrumentation during supportive periodontal therapy and concluded that there was an equal reduction in periodontal pathogens and probing pocket depth in both groups.

In deciding between dental prophylaxis devices like the Mectron Combi Touch and the EMS Prophylaxis Master, several factors require considerations. Both offer advanced features to enhance patient care and efficiency. First, assess clinical needs and services offered. The Mectron Combi Touch provides versatility with ultrasonic scaling, polishing, and air polishing functions, catering to various procedures. The EMS Prophylaxis Master excels in airflow technology, ideal for stain removal and biofilm management. Second, evaluate technological features and how they align with practice. The Mectron Combi Touch offers customizable settings and a user-friendly interface, while the EMS Prophylaxis Master focuses on precise powder delivery and ergonomic design. Patient comfort and safety are paramount; consider noise levels, vibration, and aerosol reduction features. Both prioritize these aspects, but differences may exist. Review training and support availability from manufacturers. Both Mectron and EMS offer comprehensive support, but availability may vary [40].

The choice between these devices ultimately depends on the specific needs and preferences of the dental professional and the requirements of the individual patient. Factors such as treatment goals, patient comfort, and clinical indications should be carefully considered when selecting the appropriate device for a particular case [41].

Looking ahead, the future perspectives of utilizing the Mectron Combi Touch and the EMS Prophylaxis Master extend beyond their current applications [42]. These innovative devices hold promise in evaluating additional indices beyond traditional measures, potentially revolutionizing preventive dental care. Furthermore, there is a growing need to test these devices on diverse populations, including pediatric patients, to ensure their efficacy and safety across different age groups [43, 44]. Moreover, exploring the integration of adjuvant systems in nonsurgical periodontal therapy [45, 46] alongside these devices presents an exciting avenue for enhancing treatment outcomes and patient satisfaction. By expanding their utility in these areas, the Mectron Combi Touch and the EMS Prophylaxis Master can continue to advance the field of dental prophylaxis and contribute to improved oral health outcomes for patients of all ages. Furthermore, implementing other adjuvant products and recently tested technology in combination with the tested instrumentations could provide novel insights into periodontal research [47–50].

As limitations of the study, the fact that the personal perception of patients implied the calculation of subjective outcomes. Subsequently, the fact that different operators were involved reduces the strength of the results. Further studies will aim to evaluate other periodontal parameters to correlate higher scores of probing depths to higher sensitivity during the procedures.

While there is a considerable body of literature on dental prophylaxis techniques, including air polishing and ultrasonic scaling, there is a notable lack of studies directly comparing the Mectron Combi Touch and the EMS Prophylaxis Master. Most existing research focuses on individual devices or techniques, often exploring their efficacy in isolation rather than in direct comparison. This gap in the literature posed a challenge when attempting to place our findings within the context of previous studies. As a result, our study provides a unique contribution to the field by comparing these two advanced devices head-to-head. The absence of similar comparative studies further underscores the value of our research, as it offers new insights into the effectiveness and user experience of both devices, which can help inform clinical decision-making and guide future studies in this area.

## 5. Conclusions

In conclusion, both the Mectron Combi Touch and the EMS Prophylaxis Master demonstrate comparable levels of subjective comfort for both patients and operators, with a significantly higher patient comfort for the airflow variable for the Mectron Combi Touch and a significantly higher patient comfort for the US variable for the EMS Prophylaxis Master. Regarding the periodontal parameters, they exhibited similar effectiveness in reducing periodontal parameters. These findings suggest that both devices offer viable options for dental professionals seeking efficient and patient-friendly methods for periodontal treatment.

Future research could explore the performance of these devices in different patient demographics or evaluate their effectiveness over longer treatment periods, contributing to the evolution of periodontal care.

## Figures and Tables

**Table 1:** Inclusion and exclusion criteria.

Inclusion criteria	• Age ≥18 years. • Patients who have not received dental hygiene treatment for at least 6 months before enrollment. • Periodontal disease in maintenance therapy: grading A or B and staging I–III.

Exclusion criteria	• Neurologic, psychiatric, and mental diseases. • Respiratory disorders (i.e., asthma, active respiratory infections, cystic fibrosis, etc.). • Patients taking antibiotics during the study. • Pregnant and breastfeeding women. • Patients undergoing anticancer treatment.

**Table 2:** Methods for periodontal indexes calculation.

Periodontal index	Calculation
Bleeding on probing (BoP) [18]	The number of sites where bleeding is recorded is divided by the total number of available sites in the mouth and multiplied by 100 to express the bleeding index as a percentage.

Bleeding score (BS) [19]	Presence of BoP on a scale from 0 to 3: • 0: Normal gingiva; no inflammation; no discoloration (erythema); no bleeding. • 1: Mild inflammation; slight erythema; minimal superficial alterations. No bleeding. • 2: Moderate inflammation; erythema; BoP. • 3: Severe inflammation; severe erythema and swelling; tendency to spontaneous bleeding; possible ulceration.

Plaque control record (PCR) [20]	The number of sites where plaque is recorded is divided by the total number of available sites in the mouth and multiplied by 100 to express the PCR as a percentage.

**Table 3:** Characteristics of the devices used in the study extracted from the technical data sheets of the instruments.

Device	Manufacturer	General features	Technical features
Mectron Combi Touch	Mectron S.p.a., Carasco, Italy	Combines an ultrasound device and airflow in a single unit, allowing you to perform a complete prophylaxis ablation of supra- and subgingival tartar, removal of discolorations and biofilm, and implant cleaning.	Power voltage: 100–240 Vac 50/60 Hz Waterpower: operating pressure from 1 to 6 bar Airpower: input pressure from 4 to 8 bar Power levels from 1 to 5 Device weights and dimensions: 4.8 kg weight, *l–w–h* 410 × 260 × 145 mm (without handpieces).

EMS Prophylaxis Master	EMS, Chemin de la Vuarpillière, Nyon, Switzerland	A solution for prevention and prophylaxis of gingivitis and periodontitis. Designed for intensive professional use, the device is characterized by ergonomics, high precision, ease of maintenance, and maintenance and compliance with the highest hygiene standards.	Supply voltage: 100–240 VAC 50/60Hz Waterpower: 2–5 bar (200–500 kPa) Compressed air power: 4.5–7 bar (450– 700 kPa) Power levels 1–10 Dimensions and weight: width 26 cm, depth 29 cm, height 24.5 cm, weight 5 kg.

**Table 4:** Descriptive statistics of primary outcomes represented by VAS scale.

	Mectron Combi Touch					EMS Prophylaxis Master					
Outcome	Mean	St Dev	Min	Median	Max	Mean	St Dev	Min	Median	Max	*p* Value
Sensitivity	2.65	2.60	0.00	2.50	8.00	3.30	2.62	0.00	3.00	9.00	0.365
Pain	3.25	2.71	0.00	2.50	10.00	3.70	2.75	0.00	3.50	8.00	0.379
Airflow comfort	8.70	1.66	5.00	9.50	10.00	7.55	1.28	5.00	7.00	10.00	0.045*⁣*^*∗*^
US comfort	7.40	1.14	5.00	7.50	9.00	8.30	1.22	5.00	8.00	10.00	0.027*⁣*^*∗*^

Abbreviations: US, ultrasound; VAS, visual analog scale.

*⁣* ^*∗*^ *p* < 0.05.

**Table 5:** Descriptive statistics of bleeding on probing (BoP).

Group	Time	Mean	St Dev	Min	Median	Max	Intragroup *p* value	Intergroup *p* value
Mectron	T0	21.05	9.24	5.00	21.50	40.00	—	T0 vs. T0 =
	T1	17.10	8.18	5.00	20.00	35.00	0.0001*⁣*^*∗*^	0.956
EMS	T0	19.70	7.31	10.00	20.00	40.00	—	T1 vs. T1 =
	T1	15.95	5.49	8.00	17.00	26.00	0.011*⁣*^*∗*^	0.953

*⁣* ^*∗*^ *p* < 0.05.

**Table 6:** Descriptive statistics of plaque control record (PCR).

Group	Time	Mean	St Dev	Min	Median	Max	Intragroup *p* value	Intergroup *p* value
Mectron	T0	78.90	22.04	35.00	80.00	100.00	—	T0 vs. T0 =
	T1	51.40	16.93	20.00	50.00	85.00	<0.0001*⁣*^*∗*^	0.110
EMS	T0	76.55	23.35	35.00	73.00	100.00	—	T1 vs. T1 =
	T1	46.80	14.70	20.00	46.00	85.00	<0.0001*⁣*^*∗*^	0.052

*⁣* ^*∗*^ *p* < 0.05.

**Table 7:** Descriptive statistics of the bleeding score (BS).

Group	Time	Mean	St Dev	Min	Median	Max	Intragroup *p* value	Intergroup *p* value
Mectron	T0	2.25	0.79	1.00	2.00	3.00	—	T0 vs. T0 =
	T1	1.45	0.83	0.00	2.00	3.00	<0.0001*⁣*^*∗*^	0.751
EMS	T0	2.30	0.80	1.00	2.50	3.00	—	T1 vs. T1 =
	T1	1.35	0.75	0.00	1.50	2.00	< 0.0001*⁣*^*∗*^	0.484

*⁣* ^*∗*^ *p* < 0.05.

## Data Availability

The data that support the findings of this study are available from the corresponding author upon reasonable request.
